# Radiation Oncology Physician Practice in the Modern Era: A Statewide Analysis of Medicare Reimbursement

**DOI:** 10.7759/cureus.1192

**Published:** 2017-04-25

**Authors:** Johnny Kao, Amanda Zucker, Elizabeth A Mauer, Andrew T Wong, Paul Christos, Josephine Kang

**Affiliations:** 1 Radiation Oncology, Good Samaritan Hospital Medical Center; 2 Division of Biostatistics and Epidemiology, Department of Healthcare Policy and Research, New York-Presbyterian/Weill Cornell Medical Center; 3 Radiation Oncology, New York-Presbyterian/Weill Cornell Medical Center

**Keywords:** medicare, reimbursement, radiation oncology, new york state

## Abstract

**Introduction:**

In recent years, major changes in health care policy have affected oncology practice dramatically. In this context, we examined the effect of practice structure on volume and payments for radiation oncology services using the 2013 Medicare Provider Utilization and Payment Data: Physician and Other Supplier Public Use File (POSPUF) for New York State radiation oncologists.

**Methods:**

The Medicare POSPUF data was queried, and individual physicians were classified into freestanding office-based and hospital-based practices. Freestanding practices were further subdivided into urology, hematology-oncology, and other ownership structures. Additional variables analyzed included gender, year of medical school graduation, and Herfindahl-Hirschman Index (HHI). Statistical analyses were performed to assess the impact of the above-mentioned variables on reimbursements.

**Results:**

There were 236 New York State radiation oncologists identified in the 2013 Medicare POSPUF dataset, with a total reimbursement of $91,525,855. Among freestanding centers, the mean global Medicare reimbursement was $832,974. Global Medicare reimbursement was $1,328,743 for urology practices, compared to $754,567 for hematology-oncology practices and $691,821 for other ownership structures (p < 0.05). The mean volume of on-treatment visits (OTVs) was 240.5 per year, varying by practice structure. The mean annual OTV volumes for urology practices, hematology-oncology practices, other freestanding practices, and hospital-based programs were 424.6, 311.5, 247.5, and 209.3, respectively. After correcting for gender, physician experience, and HHI, practice structure was predictive of freestanding reimbursement and on treatment visit volume.

**Conclusion:**

Higher Medicare payment was significantly predicted by the type of practice structure, with urology-based and hematology-oncology practices accounting for the highest total reimbursement and OTV volume.

## Introduction

Radiation therapy represents one of the three pillars of cancer treatment. With the rapidly aging population, the demand for radiation therapy is projected to increase significantly [1]. Broad adoption of highly complex techniques in radiation oncology has resulted in less toxicity and/or improved efficacy at a higher cost [2-4]. Prior national level analyses evaluating practice patterns identified that practice type, geography, and individual physicians accounted for the majority of variation in radiation therapy cost [5-7].

Economic constraints, competition, growing administrative burden, and proliferation of cost-containment programs have been cited as pressures on independent oncology practices that are driving practice consolidation [8]. While the effect of combined urology and radiation oncology practices on intensity modulated radiation therapy (IMRT) utilization for prostate cancer has been extensively studied, other practice structures are less well characterized [9-11].

The 2014 release of the Medicare Provider Utilization and Payment Data: Physician and Other Supplier Public Use File (POSPUF) allows for unprecedented opportunity to investigate factors associated with healthcare expenditure attributable to radiation therapy. The POSPUF datafile contains information on nearly all Medicare Part B services provided by individual physicians. Analysis of Medicare spending using POSPUF data has been detailed for multiple specialties, including ophthalmology, plastic surgery, urology, general surgery, and radiation oncology [12-15]. However, to our knowledge, an analysis of Medicare claims data by radiation oncology practice structure has not been previously reported.

Since Medicare accounts for approximately 50% of all patients diagnosed with cancer [16], we performed this analysis of Medicare claims data for New York State radiation oncologists stratified by practice setting, to determine provider and practice variables that may account for differences in utilization.

## Materials and methods

### Data source

We queried the 2013 Medicare Provider and Utilization and Payment Data: POSPUF for radiation oncologists in New York State, obtained from www.CMS.gov. These files contain a comprehensive summary of Medicare Part B payments to individual physicians. To protect patient confidentiality, billing codes of procedures performed on fewer than 10 patients per physician were embargoed. Data from these files include billing codes, the location of service, total submitted services, and total payment amount. This study was deemed to be exempt from review by the Institutional Review Board.

### Radiation oncologists

Quality assurance and post-processing of the raw data were performed to ensure that all analyzed physicians were accurately classified as practicing New York State radiation oncologists. We identified several radiation oncologists, misclassified as diagnostic radiologists and hematology oncologists and vice versa. Additionally, several physicians, classified as New York-based radiation oncologists, practice other specialties or worked in another state. Physicians who were known to have changed practices in 2013 were excluded. These physicians were often identified as having a large variation in Medicare reimbursement, compared to 2012. Physicians with less than $20,000 in Medicare Part B payments were presumed to be part-time and excluded from the analysis. A total of 236 radiation oncologists in 88 practices met the inclusion criteria for this analysis.

### Practice types

Practices were grouped into freestanding practices that billed according to the Medicare Physician Fee Schedule (MPFS) and hospital practices that billed through the hospital outpatient prospective payment system (HOPPS). A small cohort of physicians worked in practices that billed through both MPRS and HOPPS.

Among practices that billed MPFS, we considered three subgroups, including majority urology practices, majority hematology-oncology practices, and “other” practice structures, which included traditional single specialty radiation oncology practices, multispecialty practice (dominated by specialties other than urology or hematology-oncology), and national radiation oncology chains. Among practices that billed HOPPS, no attempt was made to separate out programs by type of institution.

### Radiation oncology-related procedures

Treatment codes for radiation treatment delivery, simulation, planning, weekly treatment management, brachytherapy, and stereotactic radiation were extracted (Table 1). For each code, the total number of procedures and number of patients treated were recorded. We did not independently analyze billing codes related to treatment devices, image-guidance, administration of unsealed radioactive sources or evaluation and management billing codes, although these were included in the total Medicare payments. There were no proton centers in New York State in 2013.

**Table 1:** Medicare codes used to characterize clinical activity

Code	Description
77418	Intensity modulated radiation treatment delivery
77412-77416	Radiation treatment delivery, three or more treatment areas
77407-77411	Radiation treatment delivery, two treatment areas, three or more ports
77402-77406	Radiation treatment delivery, single treatment area
77301	Management of modulation radiotherapy planning
77295	Management of radiation therapy, 3D
77290	Management of radiation therapy, simulation, complex
77427	Radiation treatment management, five treatments
77431	Radiation treatment management, one or two treatments
77432	Stereotactic radiation treatment management of brain lesions, complete course of treatment consisting of one session
77435	Stereotactic radiation treatment management of one or more lesions using imaging guidance
G0340	Image-guided robotic linear accelerator-based stereotactic radiosurgery, delivery including collimator changes and custom plugging, fractionated treatment, all lesions, per session, second through fifth sessions, maximum five sessions per course of treatment
G0339	Image-guided robotic linear accelerator-based stereotactic radiosurgery, complete course of therapy in one session or first session of fractionated treatment
77785	High dose brachytherapy delivery, one channel
77326	Brachytherapy radiation therapy plan (one to four applications, nine to 12 brachytherapy sources)
77327	Brachytherapy radiation therapy plan (five to 10 applications, nine to 12 brachytherapy sources)
77328	Brachytherapy radiation therapy plan (over 10 applications, over 12 brachytherapy sources)
55875	Insertion of needles or catheters for radiation therapy
77778	Application of radiation source, complex

### Demographic information for physicians practices

The Herfindahl-Hirschman Index (HHI) is a standard measure of economic competition that has been extensively applied to health policy research [17]. The HHI is calculated by summing the results (in percentages) of the squared market share competing firms. The maximum HHI is 10,000 (100*100) in a monopoly market and conversely approaches 0 in the setting of infinite competition. For this study, physicians were grouped into practices by billing addresses. Practices were grouped by county. The HHI was calculated by determining the market share of on-treatment visits (OTVs) per year by practice for each county. Practices were classified into above or below mean HHI. The gender of each physician was recorded and the year of medical school graduation was obtained through an online search of Vitals.com.

### Statistical analysis

One-way analysis of variance (ANOVA) tests were performed to look at differences in reimbursement and key metrics of clinical activity (total Medicare reimbursement, annual OTV volume, and IMRT utilization) between practice types and year of medical school graduation groups. Post-hoc pairwise comparisons between groups were made with Bonferroni adjustment for multiple comparisons. R-squared statistics were reported to describe the proportion of variance explained by the models. Wilcoxon rank-sum tests/independent sample t-tests were performed to look at differences in reimbursement and clinical activity by gender and HHI category (i.e., divided into above and below the mean), as appropriate.

Three multivariable generalized estimating equations (GEEs) models were performed for the outcomes of 1) reimbursement, 2) OTVs), and 3) IMRT utilization, using practice type, gender, year of medical school graduation, and HHI as predictors in the multivariable models. The GEE models provided robust standard errors due to the potential correlation between radiation oncologists in the same practice. Similarly, because the proportion of Medicare Advantage participants within the county could influence outcomes, subsequent GEEs for all three outcomes were constructed, clustering by county and the results served to confirm the original models with respect to practice setting [18]. Analyses for global Medicare reimbursement as an outcome excluded physicians in any hospital-based practice. All p-values are two-sided with statistical significance evaluated at the 0.05 alpha level. All analyses were performed in Stata Version 14.0 (StataCorp, College Station, TX) and R 3.3.1 (R Core Team, Vienna, Austria).

## Results

A total of 236 New York State radiation oncologists were identified, with a total payment of $91,525,855 from Medicare. Of this group, 176 were males, and 60 were females. The average year of medical school graduation was 1989 (range 1954–2007) (Table 2).

**Table 2:** Practice and physician characteristics

Characteristic	Number	Percentage
Physicians by Practice Type		
Urology (freestanding)	18	8%
Hematology-Oncology (freestanding)	13	6%
Other (freestanding)	56	24%
Hospital-based	137	58%
Both freestanding and hospital-based	12	5%
Practices by Ownership Structure		
Urology (freestanding)	5	6%
Hematology-Oncology (freestanding)	5	6%
Other (freestanding)	31	35%
Hospital-based	45	51%
Both freestanding and hospital-based	2	2%
Gender		
Male	176	75%
Female	60	25%
Year of Medical School Graduation		
Before 1978	39	17%
1978 to 1987	64	27%
1988 to 1997	65	28%
1998 to 2007	68	29%
Herfindahl-Hirschman Index (mean = 3268)		
Below mean	165	70%
Above mean	71	30%

### Combined technical and professional reimbursement

**Figure 1:**
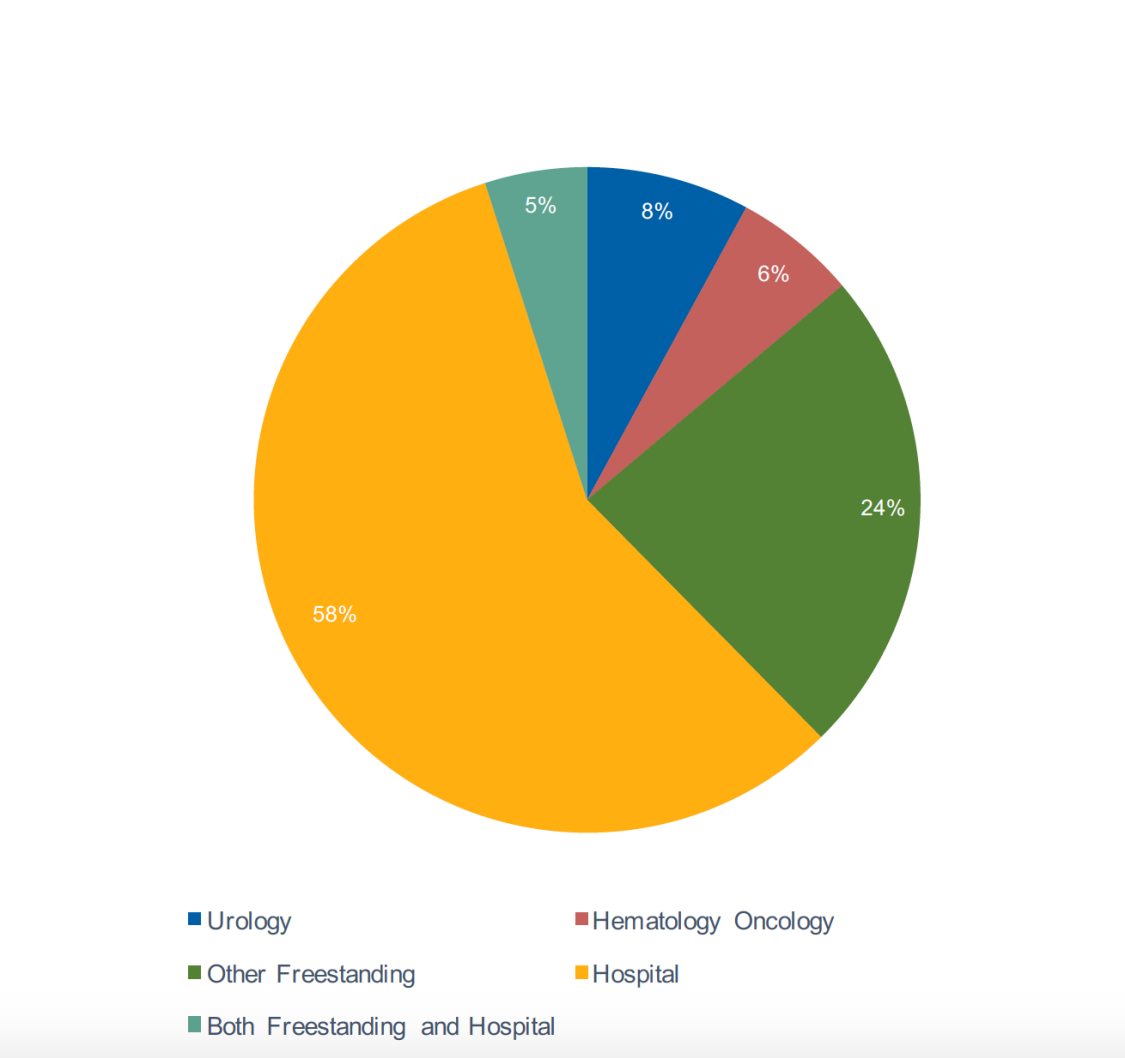
The percentage of radiation oncologists by practice type

In New York State, 37% of physicians worked for practices with global technical and professional billing. For this subset of radiation oncologists, 21% worked for urology practices, 15% worked for hematology-oncology practices, and 64% worked for other practice structures (Figure 1 and Table 2).

**Figure 2:**
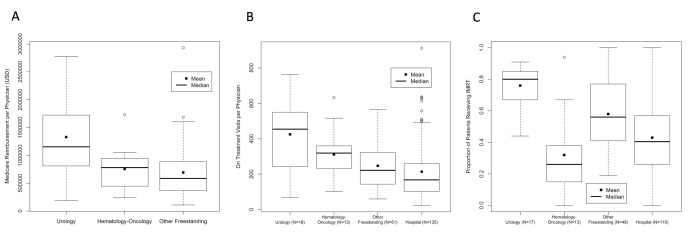
Box and whisker plot comparing radiation oncology practice types by (A) medical reimbursement per physician, (B) on treatment office visits per physician and (C) intensity modulated radiation therapy utilization

Mean global Medicare reimbursement for freestanding practices averaged $832,974 (95% confidence interval (CI) 709,269 to 956,678; median $711,586) with significant variation by practice type (Figure 2A). Urology practices averaged $1,328,743 (95% CI 974,277 to 1,683,209; median $1,153,696) compared to $754,567 (95% CI 511,421 to 997,712; median $781,528) for hematology-oncology practices and $691,821 (95% CI 558,632 to 825,010; median $585,498) for other practice structures. Reimbursement was significantly higher for urology groups compared to hematology-oncology (Bonferroni p = 0.013) or other practices (Bonferroni p < 0.001). Practice structure accounted for 19% of the observed variation in global reimbursement.

### Professional reimbursement

In New York State, 58% of physicians worked in hospital-based practices with professional billing attributed to the physician. In this structure, technical billing is performed by the institution and not available for analysis. Professional reimbursement averaged $115,588 (95% CI 102,842 to 128,334; median $100,042).

### Patient volumes across practice settings

The number of OTVs is a standard benchmark for physician productivity in radiation oncology. This metric fails to capture physician work related to stereotactic radiation, brachytherapy, image-guided radiation therapy, or IMRT. With these caveats, this is a measure of productivity for physicians working in both freestanding and hospital-based settings.

The mean number of OTVs per physician was 240.5 per year (95% CI 219.7 to 261.4; median, 196). The mean number of OTVs per physician was 424.6 per year (95% confidence 324.0 to 525.2; median 454.5) for urology practices compared to 311.5 per year (95% CI 218.4 to 404.5; median 319) for hematology-oncology practices, 247.5 per year (95% CI 211.8 to 283.1; median 222) for other freestanding practices and 209.3 per year (95% CI 183.0 to 295.6; median 168) for hospital-based programs (Figure 2B). Practice type accounted for 13% of the observed variation in OTV volumes. The volume of OTVs per physician was significantly higher for urology groups compared to other freestanding practices (Bonferroni p < 0.001) and hospital-based practices (Bonferroni p < 0.001).

### Intensity modulated radiation oncology utilization using treatment delivery codes

For freestanding centers, there are granular data on the percentage of patients receiving external radiation therapy fractions delivered via IMRT. The formula used is the number of patients with code 77418 divided by the number of patients with codes 77402 through 77418. Overall, 55% (95% CI 49% to 60%) of patients were treated with IMRT. For urology centers, 80% (95% CI 75% to 85%) of patients were treated with IMRT compared to 22% (95% CI 12% to 31%) of hematology-oncology centers and 55% (95% CI 49% to 62%) for other freestanding practices.

### Intensity modulated radiation oncology utilization using treatment planning codes

To compare IMRT utilization with hospital-based practices, we utilized IMRT planning codes that were shared across practice settings. IMRT utilization was estimated by dividing the number of patients with code 77301 divided by the number of patients with codes 77295 plus 77301. To validate the use of IMRT planning codes to estimate IMRT utilization, the Pearson’s correlation coefficient for IMRT treatment delivery vs. IMRT planning codes was 0.91 for freestanding centers. Overall, 49% (95% CI 45% to 53%) of patients were treated with IMRT. IMRT was used for 58% (95% CI 52% to 64%) of freestanding centers vs. 43% (95% CI 39% to 47%) of hospital-based centers.

For urology centers, 76% (95% CI 69% to 82%) of patients received IMRT compared to 32% (95% CI 18% to 46%) of hematology-oncology centers, 58% (95% CI 52% to 64%) of other freestanding centers and 43% (95% CI 39% to 47%) of hospital-based programs (Figure 2C). Practice type accounted for 23% of the observed variation in IMRT utilization. The percentage of patients treated with IMRT was higher for urology groups and other freestanding centers compared to hematology-oncology centers and hospitals (Bonferroni p < 0.001 for each).

### Gender, year of medical school graduation, and Herfindahl-Hirschman Index

Neither gender nor year of medical school graduation had a significant impact on global Medicare reimbursement, OTV volume, or IMRT utilization on univariate analysis (p > 0.05). Above average HHI was not associated with differences on global Medicare reimbursement or IMRT utilization but was associated with higher volumes of OTVs (p = 0.02). Physicians practicing in highly competitive markets averaged 225.4 OTV compared to 295.9 for physicians practicing in less competitive markets.

### Multivariable analysis

In multivariable modeling, urology practices demonstrated significantly greater Medicare reimbursement, compared to hematology-oncology and other freestanding practices. The biggest difference was found between urology and other freestanding practices, with physicians in urology practices receiving on average $618,746.00 more reimbursement than physicians in other freestanding practices (p < 0.001) (Table 3).

**Table 3:** Generalized estimating equation modeling Medicare reimbursement (N = 87) This model excludes physicians in any hospital-based practices.

	Estimate	Robust Std. Error	Robust z	p-value
(Intercept)	1282882.30	177138.40	7.24	<0.001
Sex, Male (ref)	-	-	-	-
Female	-61536.85	128710.00	-0.48	0.633
Practice Type, Urology (ref)	-	-	-	-
Hematology-Oncology	-526856.31	197240.70	-2.67	0.008
Other (freestanding)	-618746.02	171469.50	-3.61	<0.001
Year of Medical School Graduation, Before 1978 (ref)	-	-	-	-
1978 to 1987	67144.79	167269.50	0.40	0.688
1988 to 1997	174375.65	150083.30	1.16	0.245
1998 to 2007	89443.75	137870.00	0.65	0.516
Herfindahl-Hirschman Index (HHI), Below mean (ref)	-	-	-	-
Above mean	-141470.94	125076.90	-1.13	0.258

Similarly, urology practices demonstrated significantly greater OTV than other freestanding and hospital-based practices. The biggest difference was found between urology and hospital-based practices, with physicians in urology practices having on average 207 more OTVs than physicians in hospital-based practices (p < 0.001). Additionally, physicians with an HHI above the mean had on average 65 more OTVs than physicians with an HHI below the mean (p = 0.012) (Table 4).

**Table 4:** Generalized estimating equation modeling OTV (N = 217) This model excludes seven physicians for whom OTV values were unknown and any physician in both freestanding and hospital-based practices. OTV: On-treatment visit.

	Estimate	Robust Std. Error	Robust z	p-value
(Intercept)	398.47	52.60	7.58	<0.001
Sex, Male (ref)	-	-	-	-
Female	-35.80	20.68	-1.73	0.084
Practice Type, Urology (ref)	-	-	-	-
Hematology-Oncology	-119.98	64.61	-1.86	0.063
Other (freestanding)	-167.35	49.32	-3.39	<0.001
Hospital-based	-206.71	47.33	-4.37	<0.001
Year of Medical School Graduation, Before 1978 (ref)	-	-	-	-
1978 to 1987	14.26	34.94	0.41	0.683
1988 to 1997	25.50	32.84	0.78	0.438
1998 to 2007	4.27	33.04	0.13	0.897
Herfindahl-Hirschman Index (HHI), Below mean (ref)	-	-	-	-
Above mean	65.12	25.92	2.51	0.012

Lastly, urology practices demonstrated significantly greater IMRT utilization than hematology-oncology, other freestanding, and hospital-based practices. The biggest difference was found between urology and hematology-oncology practices, with physicians in urology practices treating on average 43% more patients with IMRT than physicians in hematology-oncology practices (p < 0.001) (Table 5).

**Table 5:** Generalized estimating equation modeling IMRT (N = 189) This model excludes 35 physicians for whom IMRT values were unknown and any physician in both freestanding and hospital-based practices. IMRT: Intensity modulated radiation therapy.

	Estimate	Robust Std. Error	Robust z	p-value
(Intercept)	76.94	5.71	13.49	<0.001
Sex, Male (ref)	-	-	-	-
Female	-1.76	3.87	-0.45	0.650
Practice Type, Urology (ref)	-	-	-	-
Hematology-Oncology	-42.65	7.59	-5.62	<0.001
Other (freestanding)	-17.53	4.59	-3.82	<0.001
Hospital-based	-32.86	3.82	-8.60	<0.001
Year of Medical School Graduation, Before 1978 (ref)	-	-	-	-
1978 to 1987	-0.26	5.33	-0.05	0.961
1988 to 1997	-0.64	5.58	-0.11	0.909
1998 to 2007	0.07	5.57	0.01	0.990
Herfindahl-Hirschman Index (HHI), Below mean (ref)	-	-	-	-
Above mean	-2.76	3.20	-0.86	0.389

### Special procedures: stereotactic radiation and brachytherapy

A total of 41 physicians treated more than 10 patients with stereotactic radiation. For high volume stereotactic radiation practices, the median number of patients treated was 21 (range 11 to 174). High volume stereotactic radiation practices were more likely to be hospital-based practices than freestanding practices (23% for hospital-based vs. 8% for freestanding, p = 0.01).

In contrast, there were only 13 physicians who treated more than 10 patients with brachytherapy. For high volume brachytherapy practices, the median number of patients treated was 16 (range 11 to 42). High volume brachytherapy practices were uncommon in both hospital-based and freestanding practices (seven percent for hospital-based vs. three percent for freestanding, p = 0.31).

## Discussion

Through the release of Medicare Provider Utilization and Payment Data, patterns of practice can be identified. Limiting the scope of the study to New York State allowed investigators to perform post-processing to classify physicians accurately to various practice structures and to enrich the database by including gender, year of medical school graduation, and Herfindahl-Hirschman Index. These efforts allowed for a more extensive and robust analysis than prior efforts [15].

We demonstrated that physicians working at urology practices generate increased revenues by combining high patient volumes with increased IMRT utilization. This report supplements and extends earlier work documenting practice patterns for combined urology and radiation oncology groups [10]. Our study confirms prior research which demonstrated that freestanding centers utilized IMRT at a higher rate than hospital-based practices [7] but provides richer detail by practice site. In addition to urology groups, our data set also explores the relative advantages and disadvantages of other practice structures at the individual physician level. The importance of competition in predicting patient volume was facilitated by use of the Herfindahl-Hirschman Index [17]. Importantly, clustering by county mitigated the potential effect of variable participation in Medicare Advantage plans [18].

While improper variation in IMRT utilization can increase costs without improving outcome, appropriate use of IMRT can be highly beneficial. For instance, a recent study demonstrated that increased use of lung IMRT reduced the likelihood of hospitalization for dehydration or pulmonary toxicity [19]. Improving normal tissue dose distributions beyond three-dimensional conformal radiotherapy clearly contributes to reduced morbidity for breast, prostate cancer and head and neck cancer patients [4, 20-21]. IMRT is clearly justified when advanced technology enables dose escalation thereby improving locoregional tumor control [22]. Further, a recent study demonstrated that increased spending on chemotherapy and radiation for stage II-III breast cancer correlated with improved survival [23]. Finally, this analysis shows that the utilization of stereotactic radiation has surpassed brachytherapy in New York State. The rise of stereotactic radiation and the relative decline of brachytherapy has been well documented [24-25].

There are several disadvantages to inherent in claims-based datasets. Since the SEER (Surveillance, Epidemiology, and End Results) registry does not cover New York State, this analysis could not consider patient level data. Therefore, it is impossible to consider differences in patient populations treated at various practice settings. It was not feasible to systematically account for physicians working part time or those heavily engaged in research or administration. Analysis of Medicare data does not necessarily correlate to practice patterns for other payers. Importantly, this cross-sectional analysis represents a retrospective snapshot in time of a highly fluid healthcare marketplace.

Although the Medicare public data set did not capture low volume codes, it is important to recognize that most patients are treated with high volume codes. For instance, RT delivery to three or more areas (77412-77416) + IMRT (77418) accounts for 99.4% of external beam treatment delivery codes, suggesting that excluding low volume codes would not materially change conclusions [5].

Despite the increasing prominence of direct to patient marketing and highly integrated multispecialty group practices, successful referral-based physician practices are still judged by ability, availability, and affability [26]. Grit, motivation, reputation, and integrity are the types of personal qualities that will prove elusive for any big data approach. Finally, it would be interesting to determine if high volume physicians had better outcomes than lower volume physicians as demonstrated in previous studies [27].

## Conclusions

A deep dive into Medicare Part B provides useful insight into recent practice patterns. This information could assist physicians and administrative leaders to develop strategic plans in radiation oncology.
